# Regular exercise reduces the risk of all-cause mortality in socially isolated older adults: the Otassha Study

**DOI:** 10.3389/fpubh.2024.1344952

**Published:** 2024-07-04

**Authors:** Manami Ejiri, Hisashi Kawai, Keigo Imamura, Yoshinori Fujiwara, Kazushige Ihara, Hirohiko Hirano, Shuichi Obuchi

**Affiliations:** ^1^Tokyo Metropolitan Institute for Geriatrics and Gerontology, Tokyo, Japan; ^2^Faculty of Medicine, Hirosaki University, Aomori, Japan

**Keywords:** social isolation, exercise, all-cause mortality, older adults, cohort study

## Abstract

**Introduction:**

Social isolation is associated with increased mortality risk. On the other hand, some older adults prefer to be alone. Additionally, predictors of isolation are mostly unchanged across interventions. Therefore, knowledge of how to prevent negative health outcomes in isolation would be beneficial. One of the factors that reduces the risk of mortality is regular exercise. However, to date, no studies to our knowledge have examined whether regular exercise reduces mortality among socially isolated individuals. This study aimed to determine the effects of the combination of social isolation and regular exercise on mortality among community-dwelling older adults.

**Methods:**

This prospective cohort study was part of the larger Otassha Study of community-dwelling older adults living in Itabashi Ward, Tokyo, Japan. In October 2012, 835 individuals (males = 350, females = 485; mean age 73.1 years) completed a comprehensive baseline health survey. Individuals were considered socially isolated if their frequency of interactions with others averaged less than once per week. Regular exercise was defined as exercise performed at least twice a week. The participants were assigned to one of the following four groups: (1) not isolated with regular exercise, (2) not isolated without regular exercise, (3) isolated with regular exercise, and (4) isolated without regular exercise. All-cause mortality information was obtained from the ward office database. Follow-ups were conducted until 1 November 2020. A Cox proportional regression analysis was performed.

**Results:**

A final analysis was performed on a complete dataset of 735 participants (males = 303, females = 432; mean age 72.9 years). A total of 132 (18.0%), 426 (58.0%), 27 (3.7%), and 150 (20.4%) participants were assigned to groups 1, 2, 3, and 4, respectively. The mortality rates in groups 1, 2, 3, and 4 were 6.1%, 9.2%, 7.4%, and 19.3%, respectively. Compared with group 1, isolated individuals who did not perform regular exercise had a significantly higher mortality rate [adjusted hazard ratio (aHR), 2.48; 95% confidence interval (CI), 1.12–5.52]. However, no significant association was noted in isolated individuals who performed regular exercise (aHR, 1.25; 95% CI, 0.26–5.91).

**Conclusion:**

Regular exercise was associated with a decrease in mortality risk, regardless of social isolation status. Thus, our results indicate that encouraging isolated older adults to exercise regularly may reduce their negative health outcomes.

## 1 Introduction

Social isolation is one of the most serious problems faced by aging societies. According to the World Health Organization, social isolation is related to a lack of contact with family, friends, and others (1). The estimated prevalence of social isolation among older adults is as high as 88%, depending on the definition of isolation (2). Social isolation in old age leads to various negative health outcomes, such as anxiety and depression (3), poor sleep quality (4), disability (5), and dementia (6). One particular concern is that social isolation increases mortality rates (7, 8). A previous study reported that social isolation has a greater effect on mortality than established risk factors, such as smoking, alcohol consumption, and physical inactivity (9). Additionally, socially isolated individuals have higher suicide rates (10). Consequently, it is important to encourage older adults to maintain interactions with others and avoid the risk of social isolation.

However, the number of other individuals with whom older adults can interact decreases with age. As individuals age, their number of friends, relatives, and other social connections decreases (11), and the death of peers limits their social interactions (8). Moreover, some older adults prefer to be alone (12). A previous study revealed that solitude-seeking has more positive ramifications for older adults (13). In this case, in addition to preventing social isolation, there is a need to examine ways to maintain health even when isolated. However, evidence from this perspective is limited. Additionally, predictors of isolation, such as sex, age, and economic status, are mostly unchanged across interventions (2). Therefore, knowledge of how to prevent negative health outcomes in isolation would be beneficial.

Exercise is a lifestyle habit that reduces mortality risk. It improves physical function and quality of life, prevents frailty and sarcopenia, reduces noncommunicable chronic diseases, and reduces all-cause and cause-specific mortality, such as cardiovascular disease and cancer (14–17). Physical inactivity and sedentary behavior increase the risk of depression and all-cause and cardiovascular mortality among older adults (18, 19). Furthermore, exercise is beneficial for diseases common among older adults. Regular exercise can have anti-atherogenic effects on the vasculature, independent of its effects on traditional cardiovascular disease risk factors (20). Additionally, exercise interventions are beneficial for glycemic control and cardiovascular risk factors associated with diabetes (21). Exercise may thus reduce mortality via these mechanisms. However, to date, no studies to our knowledge have examined whether regular exercise reduces mortality, even among socially isolated individuals at a high risk of death.

Therefore, we aimed to ascertain whether regular exercise reduces the risk of mortality among socially isolated older adults living in the community. To address this question, this study aimed to determine the effects of the combination of social isolation and regular exercise on mortality among community-dwelling older adults.

## 2 Materials and methods

### 2.1 Participants

This study was part of “the Otassha Study” on community-dwelling older adults living in Itabashi Ward, an urban area in Tokyo, Japan. The Otassha Study began in October 2011 and involves ongoing annual health checkups. At the beginning of the study, we sent a mail recruitment letter to all residents aged 65–84 years who were registered in the Basic Resident Register, excluding institutionalized residents and participants from previous surveys conducted by our institute (*N* = 7,015). In 2011, 913 older adults participated in the health checkup. The health checkups included motor and cognitive functioning tests, medical interviews, and a questionnaire on daily life, such as lifestyle habits, social interactions, and emotions. We followed up with our participants via the annual health checkups, and new participants were recruited on an annual basis as they turned 65 years of age. The details of this cohort have been described previously (22). In this study, we used data obtained in 2012 as the baseline survey. The protocol was the same annually; however, the measures varied. As social isolation was assessed for the first time in 2012, the baseline for this study was set to 2012. The sample consisted of 835 individuals (males = 350, females = 485; mean age 73.1 years). The study was conducted in October 2012. Data were collected through the comprehensive health survey.

Ethical approval was granted by the Ethics Committee of the Tokyo Metropolitan Institute for Geriatrics and Gerontology (approval no.: E-35, 2012). Prior to this study, all the participants provided written informed consent. This study was conducted in accordance with the principles of the Declaration of Helsinki.

### 2.2 Measures

#### 2.2.1 Social isolation

Social isolation was defined based on the frequency of face-to-face and non-face-to-face contact (talking on the phone or via e-mail or letter) with nonresident families and friends (23, 24). The questionnaire we utilized is widely used to assess social isolation and has been reported to be associated with mortality among older adults (23). The participants were considered socially isolated if their frequency of interaction with others averaged less than once per week (23, 24).

#### 2.2.2 Regular exercise

Regular exercise was defined according to weekly exercise frequency based on a nationwide Japanese survey, the National Health and Nutrition Survey (25). Exercising twice or more a week was considered regular exercise, as it has been found that exercising at least twice a week is necessary to improve physical fitness (26).

#### 2.2.3 All-cause mortality

All-cause mortality information was obtained from 1 October 2012 to 1 November 2020, from the database administered by the ward office. This mortality information was provided through the notification of death forms for residents.

#### 2.2.4 Covariates

Sex, age, chronic diseases, and disability in instrumental activities of daily living (IADLs) were assessed as covariates. A nurse assessed whether the participants were currently being treated for one or more of five chronic diseases: hypertension, stroke, heart disease, diabetes, and cancer. We assessed IADLs using a subscale of the Tokyo Metropolitan Institute of Gerontology Index of Competence, which includes five questions on instrumental self-maintenance (27). The total number of answers concerning what the participants were unable to perform was used as the IADLs disability score (ranging from 0 [no disability] to 5).

### 2.3 Statistical analyses

Considering social isolation and regular exercise status, the participants were assigned to one of the following four groups: (1) not isolated with regular exercise, (2) not isolated without regular exercise, (3) isolated with regular exercise, and (4) isolated without regular exercise. Data on participant characteristics are presented as means and standard deviations (SDs) for continuous variables and as numbers and percentages for categorical variables.

The relationship between the combination of social isolation and regular exercise and mortality was examined using a Cox regression model with isolation and exercise (reference: not isolated with regular exercise) as independent variables. We fitted the crude and adjusted models, which were adjusted for sex, age, chronic diseases, and IADLs disability in 2012. To assess the possibility of reverse causality, participants who died during the first year were excluded from sensitivity analysis.

All statistical analyses were performed using IBM SPSS Statistics for Windows, version 27 (IBM Japan, Ltd., Tokyo, Japan). Statistical significance was set at *p* < 0.05.

## 3 Results

After excluding 100 participants with missing data, a final analysis was performed on the complete datasets available for 735 participants (males = 303, females = 432; mean age [SD], 72.9 [5.1] years). In total, 132 (18.0%), 426 (58.0%), 27 (3.7%), and 150 (20.4%) participants were assigned to group 1 (not isolated with regular exercise), group 2 (not isolated without regular exercise), group 3 (isolated with regular exercise), and group 4 (isolated without regular exercise), respectively. The baseline characteristics of the patients are shown in Table 1. Isolated participants were more likely to be male. Participants who did not exercise regularly were slightly older than those who exercised regularly. The mortality rates in groups 1, 2, 3, and 4 were 6.1%, 9.2%, 7.4%, and 19.3%, respectively.

**Table 1:** Characteristics of the participants based on social isolation and exercise status (*n* = 735).

	Not isolated with regular exercise (*n* = 132)		Not isolated without regular exercise (*n* = 426)		Isolated with regular exercise (*n* = 27)		Isolated without regular exercise (*n* = 150)	
	*n*	%	*n*	%	*n*	%	*n*	%
**Sex**								
Male	42	31.8%	162	38.0%	14	51.9%	85	56.7%
Female	90	68.2%	264	62.0%	13	48.1%	65	43.3%
**Age** (mean [SD])	72.0	(4.6)	73.1	(5.1)	71.6	(4.9)	73.4	(5.3)
**Chronic diseases**								
0	74	56.1%	209	49.1%	17	63.0%	55	36.7%
>1	58	43.9%	217	50.9%	10	37.0%	95	63.3%
**IADLs disability** (mean [SD])	0.1	(0.3)	0.1	(0.4)	0.1	(0.5)	0.2	(0.7)
**Follow-up month** (mean [SD])	92.8	(14.3)	92.1	(13.9)	89.3	(22.8)	84.8	(23.2)
**Follow-up status**								
Survived	117	88.6%	361	84.7%	23	85.2%	106	70.7%
Died	8	6.1%	39	9.2%	2	7.4%	29	19.3%
Censored^a^	7	5.3%	26	6.1%	2	7.4%	15	10.0%

^a^Censored participants include those who moved away and refused to respond to the survey during the follow-up period. IADLs, instrumental activities of daily living; SD, standard deviation.

In the crude model, isolated individuals who did not exercise regularly had a significantly higher mortality rate [hazard ratio (HR), 3.62; 95% confidence interval (CI), 1.66–7.93] compared with those who were not isolated but did exercise regularly (Table 2). However, no significant association was noted in isolated individuals who exercised regularly (HR, 1.27; 95% CI, 0.27–5.98). In the adjusted model, only isolated participants without regular exercise showed a significantly increased mortality rate (HR, 2.48; 95% CI, 1.12–5.52). No significant association was noted in isolated participants who exercised regularly (HR, 1.25; 95% CI, 0.26–5.91). After excluding older adults who died during the first year, only isolated participants without regular exercise showed a significantly increased mortality rate (HR, 2.73; 95% CI, 1.17–6.36) in the adjusted model (Table 3).

**Table 2:** Association between social isolation and exercise status and mortality (*n* = 735).

	*n*	Death	Incidence per 1,000 person-years	Crude HR (95% CI)	Adjusted HR (95% CI)
Not isolated with regular exercise	132	8	7.84	Reference	Reference
Not isolated without regular exercise	426	39	11.93	1.53 (0.71–3.28)	1.28 (0.60–2.75)
Isolated with regular exercise	27	2	9.95	1.27 (0.27–5.98)	1.25 (0.26–5.91)
Isolated without regular exercise	150	29	27.36	3.62 (1.66–7.93)	2.48 (1.12–5.52)

Adjusted for sex, age, instrumental activities of daily living, and chronic diseases. CI, confidence interval, HR, hazard ratio.

**Table 3:** Association between social isolation and exercise status and mortality after excluding older adults who died during the first 1 year of follow-up (*n* = 733).

	*n*	Death	Incidence per 1,000 person-years	Crude HR (95% CI)	Adjusted HR (95% CI)
Not isolated with regular exercise	131	7	6.87	Reference	Reference
Not isolated without regular exercise	426	39	11.93	1.75 (0.78–3.92)	1.47 (0.66–3.29)
Isolated with regular exercise	27	2	9.95	1.45 (0.30–7.00)	1.43 (0.29–6.91)
Isolated without regular exercise	149	28	26.42	4.01 (1.75–9.19)	2.73 (1.17–6.36)

Adjusted for sex, age, instrumental activities of daily living, and chronic diseases. CI, confidence interval, HR, hazard ratio.

## 4 Discussion

This study examined the effects of the combination of social isolation, which increases mortality, and regular exercise, which decreases mortality. The results showed that, compared with participants who were not isolated and did engage in regular exercise, those who were isolated without regular exercise had a higher risk of mortality, whereas those who were isolated but engaged in regular exercise did not have an increased risk of mortality. Therefore, our study indicates that regular exercise reduces the risk of mortality among socially isolated older adults living in the community.

This study revealed that regular exercise had a positive effect on mortality risk, even in isolated individuals. This result is consistent with evidence from previous studies showing that exercise reduces mortality (16, 17). In particular, isolated individuals are at a higher risk of cardiovascular disease and death from cardiovascular disease than non-isolated individuals (28–30). It is possible that death from cardiovascular disease was reduced in our study, even in isolated older adults, because exercise can have anti-atherogenic effects on the vasculature (20) and improve cardiovascular disease (15). However, we cannot speculate on these relationships because no analysis was performed on the cause of death in this study owing to the limited sample size. Future analyses based on the cause of death will further clarify the relationship between social isolation, regular exercise, and mortality.

The prevalence of social isolation in this study was approximately 25%, similar to that previously reported for older adults in Japan (31). Preventing social isolation in old age is crucial because isolation leads to negative health outcomes, including death (7, 8). However, if the negative effects of isolation can be eliminated, we may be able to achieve a society that accepts an individual’s preference for isolation. The findings of this study may help improve the health of older adults who willingly isolate themselves. Furthermore, future analyses that consider socializing preferences may deepen our understanding of this area.

This study showed that promoting regular exercise in isolated older adults may be beneficial; however, the percentage of those who engaged in regular exercise was low (15.6%) compared with that of non-isolated older adults (23.7%). Healthy lifestyle habits, such as physical activity, increase with social connectedness (32, 33); those who are isolated may be less likely to develop exercise habits through social connectedness. Although network interventions or opinion leaders are effective in promoting healthy lifestyle habits, such as physical activity (34, 35), these interventions are likely to be ineffective for isolated individuals with few social connections. However, a previous review on the determinants of exercise reported insufficient evidence (36); thus, further investigations are required to determine how to promote the acquisition of exercise habits among socially isolated older adults. The following methods should be considered to promote regular exercise among isolated individuals. First, isolated older adults who exercise should be surveyed to determine the type of exercise they practice, and the types of exercise that are easy to practice in isolation should be promoted. For example, since ball sports require a partner, isolated older adults can practice walking, strength training, and swimming instead, as they can be practiced alone. Second, a mobile phone-based intervention may be beneficial because older adults who do not like socializing may not participate in face-to-face, classroom-type exercise classes. Indeed, the World Health Organization recommends the promotion of physical activity using digital technology for older adults (37).

### 4.1 Limitations

This study had some limitations. First, regular exercise was examined only in terms of frequency, not intensity, type, or duration. Second, owing to the small number of isolated participants who exercised regularly, we did not sufficiently adjust for other factors related to mortality, such as self-rated health and depression. A stratification analysis by sex could not be performed for the same reason. Third, since there is no standardized scale for assessing social isolation, this study possibly included only one aspect of social isolation in the analysis. Finally, as this study targeted older adults living in one area of Japan, caution should be exercised in generalizing the findings to other areas. However, to our knowledge, this is the first study to show that, even among isolated older adults, engaging in regular exercise positively affected mortality, which is highly significant in today’s aging society.

### 4.2 Conclusion and implications

Regular exercise was associated with a decrease in mortality risk, regardless of social isolation status. Therefore, our results indicate that encouraging isolated older adults to exercise regularly may reduce their negative health outcomes. Further research is required on ways to maintain health even in isolation to achieve a society that allows for a diversity of socializing preferences among older adults.

## Data availability statement

The raw data supporting the conclusions of this article will be made available by the authors, without undue reservation.

## Ethics statement

The studies involving humans were approved by the Ethics Committee of the Tokyo Metropolitan Institute for Geriatrics and Gerontology. The studies were conducted in accordance with the local legislation and institutional requirements. The participants provided their written informed consent to participate in this study.

## Author contributions

ME: Conceptualization, Data curation, Formal analysis, Investigation, Writing – original draft. HK: Conceptualization, Investigation, Writing – original draft. KIm: Conceptualization, Investigation, Writing – original draft. YF: Investigation, Writing – review & editing. KIh: Investigation, Writing – review & editing. HH: Investigation, Writing – review & editing. SO: Conceptualization, Funding acquisition, Supervision, Writing – review & editing.
